# Supplementary material for: Development of a computer-aided design software for the quantitative evaluation of aesthetic damage
Source: PLoS One. 2019 Dec 18;14(12):e0226322. doi: 10.1371/journal.pone.0226322 (PMC6919621; doi:10.1371/journal.pone.0226322)
Supplement: S1 Code site — 1. (ZIP) [file pone.0226322.s001.zip › [EESC_jr][Projeto]Relatorio_07_21/reports/templates_zip/gentelella-master/vendors/select2/docs/announcements-4.0.html]

---
layout: default
title: Select2 4.0.0 Released
slug: announcements-4.0
---

# Announcing Select2 4.0.0

# Select2 4.0.0

The 4.0 release of Select2 is the result of three years of working on the
code base and watching where it needs to go. At the core, it is a full
rewrite that addresses many of the extensibility and usability problems
that could not be addressed in previous versions.

This release contains many breaking changes, but easy-upgrade paths have
been created as well as helper modules that will allow for backwards
compatibility to be maintained with past versions of Select2. Upgrading
*will* require you to read the release notes carefully, but the
migration path should be relatively straightforward. You can view a list
of the most common changes that you will need to make
in the release notes.

Below is an in-depth review of what is new in Select2, as well as some of
the major changes that have been made.

## New features

The notable features of this new release include:

- A more flexible plugin framework that allows you to override Select2 to
  behave exactly how you want it to.
- Consistency with standard `<select>` elements for all
  data adapters, removing the need for hidden `<input>`
  elements.
- A new build system that uses AMD to keep everything organized.
- Less specific selectors allowing for Select2 to be styled to fit the
  rest of your application.

## Plugin system

Select2 now provides interfaces that allow for it to be easily extended,
allowing for anyone to create a plugin that changes the way Select2 works.
This is the result of Select2 being broken into four distinct sections,
each of which can be extended and used together to create your unique
Select2.

The adapters implement a consistent interface that is documented in the
options section for adapters, allowing
you to customize Select2 to do exactly what you are looking for. Select2
is designed such that you can mix and match plugins, with most of the core
options being built as decorators that wrap the standard adapters.

## AMD-based build system

Select2 now uses an
AMD-based build system,
allowing for builds that only require the parts of Select2 that you need.
While a custom build system has not yet been created, Select2 is open
source and will gladly accept a pull request for one.

Select2 includes the minimal almond
AMD loader, but a custom `select2.amd.js` build is available
if you already use an AMD loader. The code base (available in the
`src` directory) also uses AMD, allowing you to include Select2
in your own build system and generate your own builds alongside your
existing infrastructure.

The AMD methods used by Select2 are available as
`jQuery.fn.select2.amd.define()/require()`, allowing you to use the
included almond loader. These methods are primarily used by the
translations, but they are the recommended way to access custom modules
that Select2 provides.

# Migrating from Select2 3.5

There are a few breaking changes that migrators should be aware of when
they are coming from older versions of Select2.

If you use the full build of Select2 (`select2.full.js`), you
will be automatically notified of the major breaking changes, and
compatibility modules will be used in some cases to ensure that your code
still behaves how you were expecting.

## No more hidden input tags

In past versions of Select2, an `<input type="hidden" />`
tag was recommended if you wanted to do anything advanced with Select2,
such as work with remote data sources or allow users to add their own
tags. This had the unfortunate side-effect of servers not receiving the
data from Select2 as an array, like a standard `<select>`
element does, but instead sending a string containing the comma-separated
strings. The code base ended up being littered with special cases for the
hidden input, and libraries using Select2 had to work around the
differences it caused.

In Select2 4.0, the `<select>` element supports all core
options, and support for the old
`<input type="hidden" />` has been deprecated. This means
that if you previously declared an AJAX field with some pre-selected
options that looked like…

{% highlight html linenos %}
{% endhighlight %}

It will need to be recreated as a `<select>` element with
some `<option>` tags that have `value`
attributes that match the old value.

{% highlight html linenos %}
Select2
Chosen
selectize.js
typeahead.js
{% endhighlight %}

The options that you create should have `selected="selected"`
set so Select2 and the browser knows that they should be selected. The
`value` attribute of the option should also be set to the value
that will be returned from the server for the result, so Select2 can
highlight it as selected in the dropdown. The text within the option
should also reflect the value that should be displayed by default for the
option.

## Advanced matching of searches

In past versions of Select2, when matching search terms to individual
options, which limited the control that you had when displaying results,
especially in cases where there was nested data. The `matcher`
function was only given the individual option, even if it was a nested
options, without any context.

With the new matcher function, only the root-level options are matched and
matchers are expected to limit the results of any children options that
they contain. This allows developers to customize how options within
groups can be displayed, and modify how the results are returned.

A function has been created that allows old-style matcher functions to be
converted to the new style. You can retrieve the function from the
`select2/compat/matcher` module, which should just wrap the old
matcher function.

So if your old code used a matcher that only displayed options if they
started with the term that was entered, it would look something like…

{% highlight js linenos %}
function matchStart (term, text) {
if (text.toUpperCase().indexOf(term.toUpperCase()) == 0) {
return true;
}
return false;
}
$("select").select2({
matcher: matchStart
})
{% endhighlight %}

Then in Select2 4.0, you would need to wrap the `matchStart`
method (or the name of the matcher you created) with a
`oldMatcher` method that we have created.

{% highlight js linenos %}
function matchStart (term, text) {
if (text.toUpperCase().indexOf(term.toUpperCase()) == 0) {
return true;
}
return false;
}
$.fn.select2.amd.require(['select2/compat/matcher'], function (oldMatcher) {
$("select").select2({
matcher: oldMatcher(matchStart)
})
});
{% endhighlight %}

This will work for any matchers that only took in the search term and the
text of the option as parameters. If your matcher relied on the third
parameter containing the jQuery element representing the original
`<option>` tag, then you may need to slightly change
your matcher to expect the full JavaScript data object being passed in
instead. You can still retrieve the jQuery element from the data object
using the `data.element` property.

## More flexible placeholders

In the most recent versions of Select2, placeholders could only be
applied to the first (typically the default) option in a
`<select>` if it was blank. The
`placeholderOption` option was added to Select2 to allow users
using the `select` tag to select a different option, typically
an automatically generated option with a different value.

The `placeholder` option can now take an object as well as just
a string. This replaces the need for the old
`placeholderOption`, as now the `id` of the object
can be set to the `value` attribute of the
`<option>` tag.

For a select that looks like the following, where the first option (with a
value of `-1`) is the placeholder option…

{% highlight html linenos %}
Select an option
Something else
{% endhighlight %}

You would have previously had to get the placeholder option through the
`placeholderOption`, but now you can do it through the
`placeholder` option by setting an `id`.

{% highlight js linenos %}
$("select").select2({
placeholder: {
id: "-1",
placeholder: "Select an option"
}
})
{% endhighlight %}

And Select2 will automatically display the placeholder when the value of
the select is `-1`, which it will be by default. This does not
break the old functionality of Select2 where the placeholder option was
blank by default.

## Display reflects the actual order of the values

In past versions of Select2, choices were displayed in the order that
they were selected. In cases where Select2 was used on a
`<select>` element, the order that the server received
the selections did not always match the order that the choices were
displayed, resulting in confusion in situations where the order is
important.

Select2 will now order selected choices in the same order that will be
sent to the server.

## Changed method and option names

When designing the future option set for Select2 4.0, special care was
taken to ensure that the most commonly used options were brought over.
For the most part, the commonly used options of Select2 can still be
referenced under their previous names, but there were some changes which
have been noted.

### Removed the requirement of `initSelection`

In the past, whenever you wanted to use a custom data adapter, such as
AJAX or tagging, you needed to help Select2 out in determining the initial
values that were selected. This was typically done through the
`initSelection` option, which took the underlying data of the
input and converted it into data objects that Select2 could use.

This is now handled by
the data adapter in the
`current` method, which allows Select2 to convert the currently
selected values into data objects that can be displayed. The default
implementation converts the text and value of `option` elements
into data objects, and is probably suitable for most cases. An example of
the old `initSelection` option is included below, which
converts the value of the selected options into a data object with both
the `id` and `text` matching the selected value.

{% highlight js linenos %}
{
initSelection : function (element, callback) {
var data = [];
$(element.val()).each(function () {
data.push({id: this, text: this});
});
callback(data);
}
}
{% endhighlight %}

When using the new `current` method of the custom data adapter,
**this method is called any time Select2 needs a list** of
the currently selected options. This is different from the old
`initSelection` in that it was only called once, so it could
suffer from being relatively slow to process the data (such as from a
remote data source).

{% highlight js linenos %}
$.fn.select2.amd.require([
'select2/data/array',
'select2/utils'
], function (ArrayData, Utils) {
function CustomData ($element, options) {
CustomData.\_\_super\_\_.constructor.call(this, $element, options);
}
Utils.Extend(CustomData, ArrayData);
CustomData.prototype.current = function (callback) {
var data = [];
var currentVal = this.$element.val();
if (!this.$element.prop('multiple')) {
currentVal = [currentVal];
}
for (var v = 0; v < currentVal.length; v++) {
data.push({
id: currentVal[v],
text: currentVal[v]
});
}
callback(data);
};
$("#select").select2({
dataAdapter: CustomData
});
}
{% endhighlight %}

The new `current` method of the data adapter works in a similar
way to the old `initSelection` method, with three notable
differences. The first, and most important, is that **it is called
whenever the current selections are needed** to ensure that Select2
is always displaying the most accurate and up to date data. No matter
what type of element Select2 is attached to, whether it supports a
single or multiple selections, the data passed to the callback
**must be an array, even if it contains one selection**.
The last is that there is only one parameter, the callback to be
executed with the latest data, and the current element that Select2 is
attached to is available on the class itself as
`this.$element`.

If you only need to load in the initial options once, and otherwise will
be letting Select2 handle the state of the selections, you don't need to
use a custom data adapter. You can just create the
`<option>` tags on your own, and Select2 will pick up
the changes.

{% highlight js linenos %}
var $element = $('select').select2(); // the select element you are working with
var $request = $.ajax({
url: '/my/remote/source' // wherever your data is actually coming from
});
$request.then(function (data) {
// This assumes that the data comes back as an array of data objects
// The idea is that you are using the same callback as the old `initSelection`
for (var d = 0; d < data.length; d++) {
var item = data[d];
// Create the DOM option that is pre-selected by default
var option = new Option(item.text, item.id, true, true);
// Append it to the select
$element.append(option);
}
// Update the selected options that are displayed
$element.trigger('change');
});
{% endhighlight %}

### Custom data adapters instead of `query`

In the past, any time
you wanted to hook Select2 up to a different data source you would be
required to implement custom `query` and
`initSelection` methods. This allowed Select2 to determine the
initial selection and the list of results to display, and it would handle
everything else internally, which was fine more most people.

The custom `query` and `initSelection` methods have
been replaced by
custom data adapters that handle
how Select2 stores and retrieves the data that will be displayed to the
user. An example of the old `query` option is provided below,
which is
the same as the old example,
and it generates results that contain the search term repeated a certain
number of times.

{% highlight js linenos %}
{
query: function (query) {
var data = {results: []}, i, j, s;
for (i = 1; i < 5; i++) {
s = "";
for (j = 0; j < i; j++) {s = s + query.term;}
data.results.push({id: query.term + i, text: s});
}
query.callback(data);
}
}
{% endhighlight %}

This has been replaced by custom data adapters which define a similarly
named `query` method. The comparable data adapter is provided
below as an example.

{% highlight js linenos %}
$.fn.select2.amd.require([
'select2/data/array',
'select2/utils'
], function (ArrayData, Utils) {
function CustomData ($element, options) {
CustomData.\_\_super\_\_.constructor.call(this, $element, options);
}
Utils.Extend(CustomData, ArrayData);
CustomData.prototype.query = function (params, callback) {
var data = {
results: []
};
for (var i = 1; i < 5; i++) {
var s = "";
for (var j = 0; j < i; j++) {
s = s + params.term;
}
data.results.push({
id: params.term + i,
text: s
});
}
callback(data);
};
$("#select").select2({
dataAdapter: CustomData
});
}
{% endhighlight %}

The new `query` method of the data adapter is very similar to
the old `query` option that was passed into Select2 when
initializing it. The old `query` argument is mostly the same as
the new `params` that are passed in to query on, and the
callback that should be used to return the results is now passed in as the
second parameter.

### Renamed templating options

Select2 previously provided multiple options for formatting the results
list and selected options, commonly referred to as "formatters", using the
`formatSelection` and `formatResult` options. As the
"formatters" were also used for things such as localization,
which has also changed, they have been
renamed to `templateSelection` and `templateResult`
and their signatures have changed as well.

You should refer to the updated
documentation on templates when
migrating from previous versions of Select2.

### The `id` and `text` properties are strictly enforced

When working with array and AJAX data in the past, Select2 allowed a
custom `id` function or attribute to be set in various places,
ranging from the initialization of Select2 to when the remote data was
being returned. This allowed Select2 to better integrate with existing
data sources that did not necessarily use the `id` attribute to
indicate the unique identifier for an object.

Select2 no longer supports a custom `id` or `text`
to be used, but provides integration points for converting incorrect data
to the expected format.

#### When working with array data

Select2 previously supported defining array data as an object that matched
the signature of an AJAX response. A `text` property could be
specified that would map the given property to the `text`
property on the individual objects. You can now do this when initializing
Select2 by using the following jQuery code to map the old
`text` and `id` properties to the new ones.

{% highlight js linenos %}
var data = $.map([
{
pk: 1,
word: 'one'
},
{
pk: 2,
word: 'two'
}
], function (obj) {
obj.id = obj.id || obj.pk;
obj.text = obj.text || obj.word;
return obj;
});
{% endhighlight %}

This will result in an array of data objects that have the `id`
properties that match the existing `pk` properties and
`text` properties that match the existing `word`
properties.

#### When working with remote data

The same code that was given above can be used in the
`processResults` method of an AJAX call to map properties there
as well.

### Renamed translation options

In previous versions of Select2, the default messages provided to users
could be localized to fit the language of the website that it was being
used on. Select2 only comes with the English language by default, but
provides
community-contributed translations for
many common languages. Many of the formatters have been moved to the
`language` option and the signatures of the formatters have
been changed to handle future additions.

### Declaring options using `data-*` attributes

In the past, Select2 has only supported declaring a subset of options
using `data-*` attributes. Select2 now supports declaring all
options using the attributes, using
the format specified in the documentation.

You could previously declare the URL that was used for AJAX requests using
the `data-ajax-url` attribute. While Select2 still allows for
this, the new attribute that should be used is the
`data-ajax--url` attribute. Support for the old attribute will
be removed in Select2 4.1.

Although it was not documented, a list of possible tags could also be
provided using the `data-select2-tags` attribute and passing in
a JSON-formatted array of objects for tags. As the method for specifying
tags has changed in 4.0, you should now provide the array of objects using
the `data-data` attribute, which maps to
the array data option. You should also
enable tags by setting `data-tags="true"` on the object, to
maintain the ability for users to create their own options as well.

If you previously declared the list of tags as…

{% highlight html linenos %}
{% endhighlight %}

…then you should now declare it as…

{% highlight html linenos %}
{% endhighlight %}

## Deprecated and removed methods

As Select2 now uses a `<select>` element for all data
sources, a few methods that were available by calling
`.select2()` are no longer required.

### .select2("val")

The `"val"` method has been deprecated and will be removed in
Select2 4.1. The deprecated method no longer includes the
`triggerChange` parameter.

You should directly call `.val` on the underlying
`<select>` element instead. If you needed the second
parameter (`triggerChange`), you should also call
`.trigger("change")` on the element.

{% highlight js linenos %}
$("select").val("1").trigger("change"); // instead of $("select").select2("val", "1");
{% endhighlight %}

### .select2("enable")

Select2 will respect the `disabled` property of the underlying
select element. In order to enable or disable Select2, you should call
`.prop('disabled', true/false)` on the
`<select>` element. Support for the old methods will be
completely removed in Select2 4.1.

{% highlight js linenos %}
$("select").prop("disabled", true); // instead of $("select").enable(false);
{% endhighlight %}

{% include nav/announcements-4.0.html %}
